# Complete Genome Sequence of a Bacteriophage, pVco-5, That Infects *Vibrio coralliilyticus*, Which Causes Bacillary Necrosis in Pacific Oyster (*Crassostrea gigas*) Larvae

**DOI:** 10.1128/genomeA.01143-17

**Published:** 2018-01-11

**Authors:** Hyoun Joong Kim, Jin Woo Jun, Sib Sankar Giri, Cheng Chi, Saekil Yun, Sang Guen Kim, Sang Wha Kim, Jeong Woo Kang, Se Jin Han, Se Chang Park

**Affiliations:** a Laboratory of Aquatic Biomedicine, College of Veterinary Medicine and Research Institute for Veterinary Science, Seoul National University, Seoul, Republic of Korea

## Abstract

We report here the complete genome sequence of the *Vibrio coralliilyticus*-specific phage pVco-5, a double-stranded DNA virus isolated from an oyster hatchery tank. *Vibrio coralliilyticus* causes bacillary necrosis in marine bivalve larvae; hence, phage pVco-5 could be used to prevent *V. coralliilyticus* infections in these larvae.

## GENOME ANNOUNCEMENT

Since the development of artificial seed culture of marine bivalves, mass mortality due to *Vibrio coralliilyticus* infection has occurred worldwide (1–3). Excessive use of antibiotics to manage bacterial diseases can cause food and environmental pollution. Therefore, we considered phage therapy to prevent and manage *V. coralliilyticus* infection in the larvae of the Pacific oyster, *Crassostrea gigas*.

The phage pVco-5 was isolated from tank water of an oyster hatchery in Goseong, Republic of Korea. This phage is a double-stranded DNA virus belonging to the *Podoviridae* family. Phage DNA was extracted using the phenol-chloroform extraction method (4) and sequenced using the Illumina HiSeq 2500 platform at Genotech (Daejeon, Republic of Korea). In total, 15,682,948 reads (1,583,977,748 bp) were trimmed and assembled using CLC Genomics Workbench version 6.5.1 (Qiagen, Netherlands). Open reading frame (ORF) prediction and annotation were conducted using Glimmer version 3.02 (5), Prodigal version 1.20 (6), GeneMarkS version 4.08 (7), and protein BLAST (8) and confirmed by the Rapid Annotations using Subsystems Technology (RAST) version 2.0 server (9). tRNAs were predicted using tRNAscan-SE version 2.0 (10), and the nucleotide homology of pVco-5 was determined using EMBOSS Stretcher (11).

The genome of pVco-5 comprised double-stranded linear DNA of 74,325 bp and a G+C content of 38%. The genome of pVco-5 was approximately 77 to 79% homologous to that of other *Vibrio* phages, including phi 1, JSF3, VCO139, and JA-1 (sequences obtained from GenBank). Of the 125 ORFs predicted, 116 were hypothetical proteins. The remaining 9 ORFs were classified into 1 of the following 4 groups: DNA metabolism (DNA-directed RNA polymerase RNAP1 and RNAP2, DNA polymerase, and AAA domain protein), packaging (portal protein and terminase large subunit), lysis (*N*-acetylmuramoyl-l-alanine amidase), and phage structure (capsid protein). The lytic phage pVco-5 may have potential for phage therapy of *Vibrio* infections in marine bivalve larvae.

### Accession number(s).

The complete genome sequence of phage pVco-5 was deposited in GenBank under the accession number KY612839.
